# Pressure Tuning of Superconductivity of LaPt_4_Ge_12_ and PrPt_4_Ge_12_ Single Crystals

**DOI:** 10.3390/ma15082743

**Published:** 2022-04-08

**Authors:** Gustavo A. Lombardi, Kamal Mydeen, Roman Gumeniuk, Andreas Leithe-Jasper, Walter Schnelle, Ricardo D. dos Reis, Michael Nicklas

**Affiliations:** 1 Brazilian Synchrotron Light Laboratory (LNLS), Brazilian Center for Research in Energy and Materials (CNPEM), Campinas 13083-970, SP, Brazil; gustavo.lombardi@lnls.br (G.A.L.); ricardo.reis@lnls.br (R.D.d.R.); 2 Max Planck Institute for Chemical Physics of Solids, Nöthnitzer Str. 40, 01187 Dresden, Germany; kmydeenhp@gmail.com (K.M.); andreas.leithe-jasper@cpfs.mpg.de (A.L.-J.); walter.schnelle@cpfs.mpg.de (W.S.); 3 Institut für Experimentelle Physik, TU Bergakademie Freiberg, Leipziger Straße 23, 09596 Freiberg, Germany; roman.gumeniuk@physik.tu-freiberg.de

**Keywords:** superconductivity, hydrostatic pressure, bulk modulus, skutterudites

## Abstract

We carried out electrical resistivity and X-ray diffraction (XRD) studies on the filled skutterudite superconductors LaPt${}_{4}$Ge${}_{12}$ and PrPt${}_{4}$Ge${}_{12}$ under hydrostatic pressure. The superconducting transition temperature $T_{c}$ is linearly suppressed upon increasing pressure, though the effect of pressure on $T_{c}$ is rather weak. From the analysis of the XRD data, we obtain bulk moduli of B=106 GPa and B=83 GPa for LaPt${}_{4}$Ge${}_{12}$ and PrPt${}_{4}$Ge${}_{12}$, respectively. The knowledge of the bulk modulus allows us to compare the dependence of $T_{c}$ on the unit-cell volume from our pressure study directly with that found in the substitution series La${}_{1-x}$Pr${}_{x}$Pt${}_{4}$Ge${}_{12}$. We find that application of hydrostatic pressure can be characterized mainly as a volume effect in LaPt${}_{4}$Ge${}_{12}$ and PrPt${}_{4}$Ge${}_{12}$, while substitution of Pr for La in La${}_{1-x}$Pr${}_{x}$Pt${}_{4}$Ge${}_{12}$ yields features going beyond a simple picture.

## 1. Introduction

The family of filled skutterudite compounds *M*Pt${}_{4}$Ge${}_{12}$ crystallizes in the cubic LaFe${}_{4}$P${}_{12}$-type structure [1]. Depending on the filler metal ions *M* a variety of different ground states has been reported. The Pt–Ge framework is capable of incorporating the alkaline-earth metals Sr and Ba [2,3] rare-earth metals La, Ce, Pr, Nd, Sm, and Eu [3] as well as the actinides Th [4] and U [5].

NdPt${}_{4}$Ge${}_{12}$ and EuPt${}_{4}$Ge${}_{12}$ display complex magnetic phase diagrams at low temperatures [6], SmPt${}_{4}$Ge${}_{12}$ is an intermediate valence compound not showing any ordering phenomena [7,8] and CePt${}_{4}$Ge${}_{12}$ sits at the border between intermediate valence of Ce and heavy-fermion behavior [8,9,10,11]. Finally, several *M*Pt${}_{4}$Ge${}_{12}$ compounds (M=Sr, Ba, La, Pr, and Th) become superconductors with $T_{c}$ up to 8.3 K [3,12,13].

The two superconducting family members LaPt${}_{4}$Ge${}_{12}$ and PrPt${}_{4}$Ge${}_{12}$, with $T_{c}$ of 8.3 K and 7.9 K, respectively [3], have drawn a lot of attention due to their relatively high $T_{c}$ and their unusual superconducting properties. It is important to note that the Pr ion is in a singlet crystalline electric field ground state in PrPt${}_{4}$Ge${}_{12}$ and therefore nonmagnetic at low temperatures [3,12].

The nature of the superconducting order parameter in LaPt${}_{4}$Ge${}_{12}$ and PrPt${}_{4}$Ge${}_{12}$ is still under debate. In PrPt${}_{4}$Ge${}_{12}$, there are indications for the presence of point nodes in the superconducting energy-gap function from nuclear magnetic resonance (NMR) [13], specific heat and penetration depth [14] measurements. Furthermore, several physical probes suggest the multi-band character of superconductivity in PrPt${}_{4}$Ge${}_{12}$ [14,15,16,17,18,19,20,21,22]. Moreover, there is convincing evidence for time-reversal-symmetry breaking superconductivity in PrPt${}_{4}$Ge${}_{12}$ provided by muon-spin-rotation (μSR) experiments [12,23,24,25].

In LaPt${}_{4}$Ge${}_{12}$, the situation concerning the nature of the superconducting gap is less clear than in PrPt${}_{4}$Ge${}_{12}$. There is evidence for a single isotropic gap from specific heat and thermal conductivity [26], NMR [9], and photoelectron spectroscopy [15], while other studies using specific heat [19], μSR and penetration-depth measurements [27], and Fermi-surface studies [22] point at a multi-gap superconducting order parameter.

In contrast, the continuous evolution of the superconducting $T_{c}$ along the substitution series La${}_{1-x}$Pr${}_{x}$Pt${}_{4}$Ge${}_{12}$ suggests compatible order parameters of both series end compounds [12,25]. Indications for time-reversal symmetry breaking are absent in LaPt${}_{4}$Ge${}_{12}$ [12], but they are observed for Pr concentrations x≳0.5 in La${}_{1-x}$Pr${}_{x}$Pt${}_{4}$Ge${}_{12}$ [25]. What remains unclear is the origin of the pronounced minimum in $T_{c}\left(x\right)$ around x≈0.75 [12,25]. Its position does not seem to be related with the observation of the time-reversal symmetry breaking superconductivity. In contrast to $T_{c}\left(x\right)$, the unit-cell volume decreases monotonously with increasing *x* in the series in La${}_{1-x}$Pr${}_{x}$Pt${}_{4}$Ge${}_{12}$. This calls for hydrostatic pressure experiments on LaPt${}_{4}$Ge${}_{12}$ and PrPt${}_{4}$Ge${}_{12}$ to investigate the effect of a change in the unit-cell volume on the superconducting properties avoiding the complications of chemical substitution.

In the present paper, we performed an electrical resistivity and X-ray diffraction (XRD) study under hydrostatic pressure on LaPt${}_{4}$Ge${}_{12}$ and PrPt${}_{4}$Ge${}_{12}$. By combining the results from both experiments we obtain the dependence of the superconducting transition temperature on the unit-cell volume *V* of the cubic crystal structure for both compounds. This allows us to compare directly the effect of hydrostatic pressure on superconductivity in the end member compounds with that in the substitution series La${}_{1-x}$Pr${}_{x}$Pt${}_{4}$Ge${}_{12}$. We find a linear in volume dependence of $T_{c}\left(V\right)$ in our pressure study on LaPt${}_{4}$Ge${}_{12}$ and PrPt${}_{4}$Ge${}_{12}$, in contrast to the nonmonotonic dependence of $T_{c}\left(V\right)$ in the substitution series.

## 2. Experimental Details

The electrical resistivity and XRD experiments under hydrostatic pressure were carried out on single crystals of LaPt${}_{4}$Ge${}_{12}$ and PrPt${}_{4}$Ge${}_{12}$. The details of the sample preparation and characterization can be found in Ref. [28].

Four probe electrical-resistance measurements on LaPt${}_{4}$Ge${}_{12}$ and PrPt${}_{4}$Ge${}_{12}$ were carried out using an LR700 resistance bridge (Linear Research). Temperatures down to 1.8 K and magnetic fields up to 9 T were achieved in a Physical Property Measurement System (PPMS, Quantum Design). Pressures up to 2.74 GPa were generated in a double-layer piston-cylinder type pressure cell using silicon oil as pressure-transmitting medium [29]. The pressure dependence of the superconducting transition temperature of a piece of lead mounted along the whole sample space served as pressure gauge. The narrow superconducting transition width at all pressures confirmed the good hydrostatic pressure conditions inside the pressure cell.

The powder XRD data were obtained at the Extreme Methods of Analysis (EMA) beamline at the Brazilian Synchrotron Light Laboratory. The EMA beamline uses, as X-rays source, a 22 mm period Kyma undulator that delivers photons between 5 keV (3rd harmonic) and 30 keV (13th harmonic). The outgoing beam is monochromatized by a liquid N${}_{2}$ cooled high-resolution double crystal monochromator that uses two sets of Si crystals ([111] or [311]). Finally, an achromatic set of K-B mirrors focuses the beam at the sample position with a spot size down to 1.0μm×0.5μm [30]. The measurements were carried out at ambient temperature using a 20 keV (9th harmonic, λ=0.6199 Å) beam with a spot size of 15μm×15μm at the sample. The two-dimensional diffraction images were captured in a transmission geometry by a CCD MAR165 detector with pixel size of 73.2μm×73.2μm. These images were integrated in Dioptas 0.4.0 [31]. We used the NIST (National Institute of Standards and Technology, Gaithersburg, MD, USA) standard reference material 660c (LaB${}_{6}$) for calibration of detector distance and other geometrical parameters.

The single-crystalline samples were powdered and loaded each one into a diamond anvil cell (DAC) along with a ruby ball using a stainless steel gasket. The pressure was determined in situ by the wavelength of the position of the maximum of the second peak of the ruby fluorescence. We used a mixture of methanol-ethanol (4:1) as pressure transmitting medium. The pressure was controlled by a gas-membrane mechanism that was attached to the DAC.

## 3. Results

**Figure 1:**
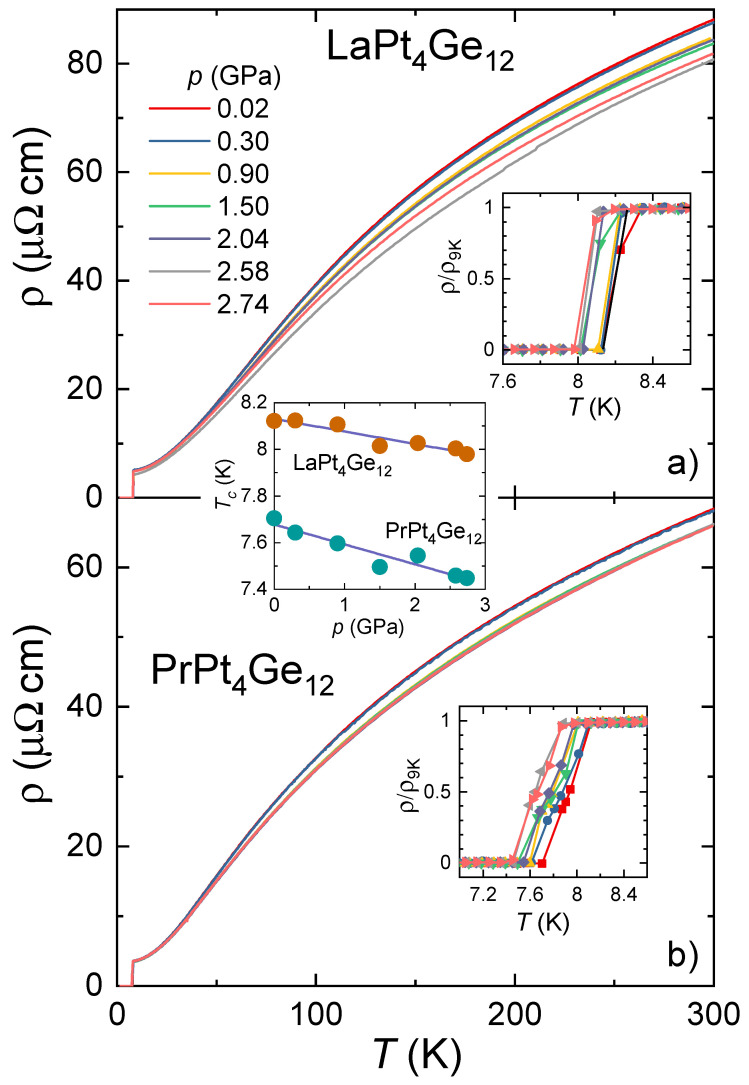
Electrical resistivity for (**a**) LaPt${}_{4}$Ge${}_{12}$ and (**b**) PrPt${}_{4}$Ge${}_{12}$ single crystals under various hydrostatic pressures. The corresponding insets depict the resistivity normalized by its value at 9 K. The central inset shows the pressure dependence of $T_{c}$. The lines are linear fits to the data. See text for details.

The temperature dependence of the electrical resistivity (ρ) for LaPt${}_{4}$Ge${}_{12}$ and PrPt${}_{4}$Ge${}_{12}$ under various hydrostatic pressures (*p*) up to 2.74 GPa is shown in Figure 1a,b. For both materials ρ(T) exhibits metallic behavior at all pressures, before a jump to zero resistance indicates the onset of superconductivity at low temperatures.

We first turn to the results on LaPt${}_{4}$Ge${}_{12}$. At ambient pressure, zero resistance is observed below $T_{c,\mathrm{zero}}=8.12$ K (inset of Figure 1a). The temperature at the midpoint of the resistive transition $T_{c,\mathrm{mid}}=8.24$ K agrees well with previous reports [3,9]. Increasing pressure leads to a decrease in the isothermal resistivity at room temperature ($\rho_{300K}$) up to 2.58 GPa before it starts to increase slightly again. $T_{c,\mathrm{zero}}\left(p\right)$ decreases only weakly with increasing pressure, by 0.14 K between ambient pressure and 2.74 GPa, the highest pressure of our investigation. Considering the scatter in the data, $T_{c}\left(p\right)$ can be described by a straight line, as depicted in the central inset of Figure 1. A linear fit to the data results in a slope of $dT_{c}\left(p\right)/dp=-53$ mK/GPa, corresponding to a normalized initial slope of $1/T_{c}\times dT_{c}/dp=\;d\left(\ln T_{c}\right)/dp=-0.0064$ GPa${}^{-1}$. We do not observe any considerable change in the width of the superconducting transition in ρ(T) in the whole pressure range.

For PrPt${}_{4}$Ge${}_{12}$, we observed ρ=0 at ambient pressure below $T_{c,\mathrm{zero}}=7.7$ K as shown in the inset of Figure 1b. Upon increasing pressure, we find a monotonous decrease in $\rho_{300K}\left(p\right)$ in the whole investigated pressure range. $T_{c,\mathrm{zero}}\left(p\right)$ exhibits a much stronger pressure dependence than for LaPt${}_{4}$Ge${}_{12}$. $T_{c,\mathrm{zero}}\left(p\right)$ drops by 0.26 K from 0 to 2.74 GPa. Considering the scatter in the data, a linear fit describes the data reasonably well and gives a slope of $dT_{c}\left(p\right)/dp=-85$ mK/GPa and $d(\ln T_{c})/dp=-0.011$ GPa${}^{-1}$, see central inset of Figure 1. We note that Foroozani et al. reported a larger slope $dT_{c}\left(p\right)/dp$ obtained from an analysis of magnetic susceptibility measurements on a polycrystal [32]. Surprisingly, the normalized initial slope for PrPt${}_{4}$Ge${}_{12}$, $d(\ln T_{c})/dp=-0.011$ GPa${}^{-1}$ is almost twice as large as for LaPt${}_{4}$Ge${}_{12}$, $d(\ln T_{c})/dp=-0.0064$ GPa${}^{-1}$.

**Figure 2:**
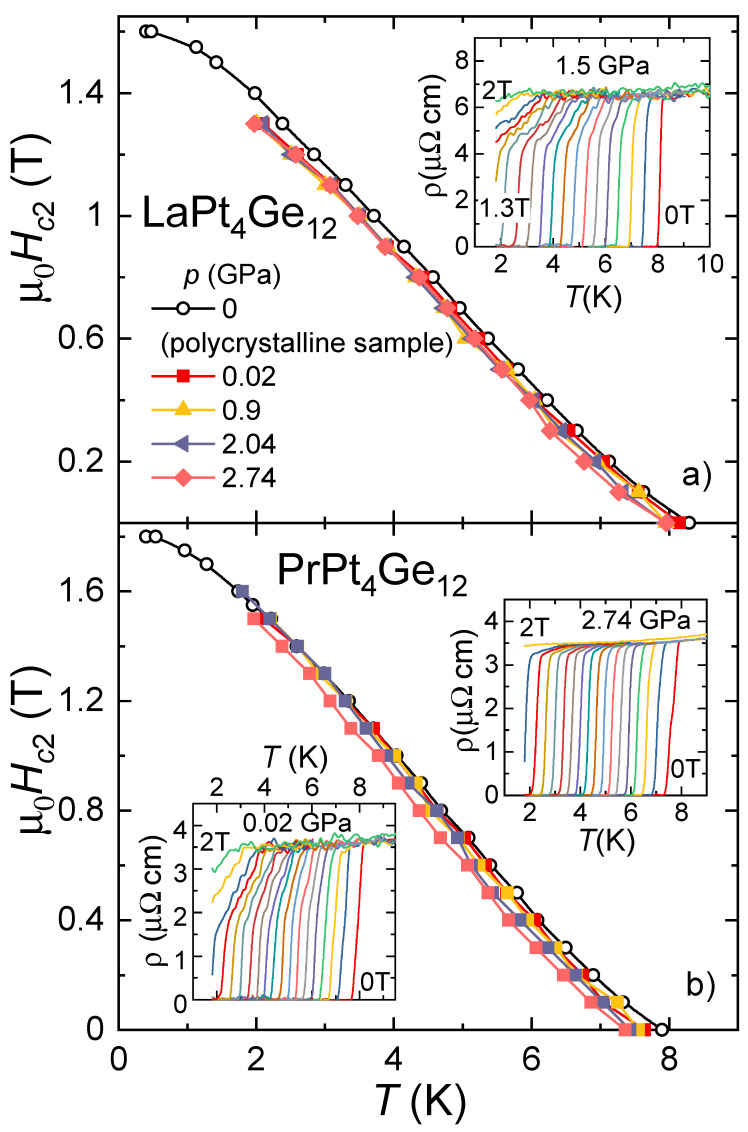
Magnetic field–temperature phase diagram of (**a**) LaPt${}_{4}$Ge${}_{12}$ and (**b**) PrPt${}_{4}$Ge${}_{12}$ single crystals at different pressures. The open symbols represent results of resistivity measurements at p=0 on polycrystalline samples down to 350 mK using the Helium-3 option of a PPMS. The lines serve as guide to the eyes. The insets show the resistivity data in different magnetic fields for selected pressures as indicated.

To determine the temperature dependence of the superconducting upper-critical field, $H_{c2}\left(T\right)$, we conducted measurements of ρ(T) in different magnetic fields for all pressures. Representative ρ(T) data are shown in the insets of Figure 2 for LaPt${}_{4}$Ge${}_{12}$ and PrPt${}_{4}$Ge${}_{12}$, respectively. The main panels display the $H_{c2}\left(T\right)$ derived from the resistivity data for selected pressures.

For LaPt${}_{4}$Ge${}_{12}$ the superconducting transition in ρ(T) gradually broadens with increasing magnetic field as shown for 1.5 GPa in the inset of Figure 2a. There is almost no difference in the $H_{c2}\left(T\right)$ curves at different pressures. In the accessible temperature range above 1.8 K, $H_{c2}\left(T\right)$ exhibits an almost linear temperature dependence only a small curvature develops at high field while a small tail is observable in $H_{c2}\left(T\right)$ close to $T_{c}$. The weak tail close to $T_{c}$ is consistent with multi-band superconductivity. An extrapolation of $H_{c2}\left(T\right)$ to zero temperature gives an upper threshold of $\mu_{0}H_{c2}^{0}\approx 1.6$ T for the upper-critical field. The pressure data are consistent with experiments on a polycrystalline sample at ambient pressure down to lower temperatures (open symbols in Figure 2a).Therefore, we may conclude that the upper-critical field, $H_{c2}^{0}$, does not change significantly with pressure in LaPt${}_{4}$Ge${}_{12}$ in the studied pressure range.

The results of the same experiments on PrPt${}_{4}$Ge${}_{12}$ are shown in Figure 2b for selected pressures. The shape of the $H_{c2}\left(T\right)$ curves is similar as described for LaPt${}_{4}$Ge${}_{12}$ above. $H_{c2}\left(T\right)$ displays a small tail close to $T_{c}$, indicative of multi-band superconductivity, and already starts to bend over at the lowest accessible temperature. The effect of pressure on $H_{c2}\left(T\right)$ is also small, but it is more pronounced than in the case of LaPt${}_{4}$Ge${}_{12}$. An upper threshold of $\mu_{0}H_{c2}^{0}\approx 1.8$ T can be estimated from an extrapolation of $H_{c2}\left(T\right)$ to zero temperature. The extrapolated value agrees well with literature [33]. ρ(T) data in different magnetic fields at a pressure of 0.02 and 2.74 GPa are shown in the lower and upper insets of Figure 2b, respectively. The broadening of the superconducting transition with increasing magnetic field observed in ρ(T) at low pressures, here 0.02 GPa is shown, is absent at 2.74 GPa. At this pressure, the transition remains sharp up to the highest field where we can access the transition in our experimental temperature range. This is remarkable since $T_{c,\mathrm{zero}}$, respectively, the $H_{c2}\left(T\right)$ curves are nearly unchanged by the application of pressure up to 2.74 GPa.

**Figure 3:**
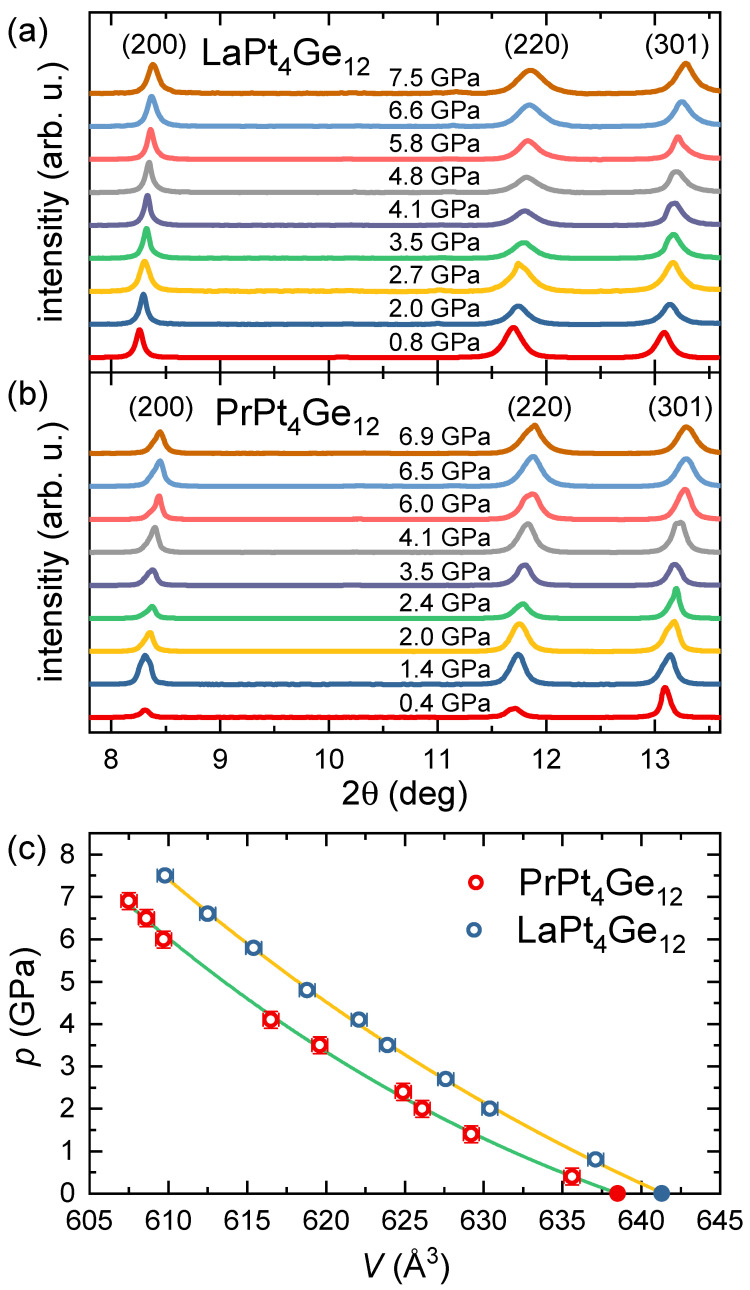
Normalized XRD data for different pressure values of (**a**) LaPt${}_{4}$Ge${}_{12}$ and (**b**) PrPt${}_{4}$Ge${}_{12}$ taken at room temperature. (**c**) Applied pressure plotted versus the unit-cell volume. The solid lines correspond to fits of the Birch-Murnaghan equation of state to the data of LaPt${}_{4}$Ge${}_{12}$ and PtPt${}_{4}$Ge${}_{12}$. The data points at p=0 (solid symbols) have been taken from literature [28]. See text for details.

Figure 3a,b shows the XRD data taken at room temperature for several pressures on LaPt${}_{4}$Ge${}_{12}$ and PrPt${}_{4}$Ge${}_{12}$, respectively. Both compounds maintain the cubic structural phase in the entire range of pressure studied up to 7.5 GPa. We can clearly identify three reflections associated with the [200], [220], and [301] planes. They are slightly shifted to higher angles upon increasing pressure, as expected due to the contraction of the crystal lattice. We used the Le-Bail method implemented in GSAS-II software package [34] to calculate the lattice parameter *a* as a function of pressure for both compounds. We note that here we only used the diffracted peak position to estimate the lattice parameters since the intensity and shape of the peak in our data are affected by the poor grain distribution due to small amount of sample in the DAC combined with small spot size of the beam.

The pressure dependence of the unit-cell volume obtained from the experimental lattice parameters of LaPt${}_{4}$Ge${}_{12}$ and PrPt${}_{4}$Ge${}_{12}$ were fitted using the Birch-Murnaghan equation of state,

$$p\left(V\right)=\frac{3B}{2}\left[{\left(\frac{V_{0}}{V}\right)}^{\frac{7}{3}}-{\left(\frac{V_{0}}{V}\right)}^{\frac{5}{3}}\right]\left\{1+\frac{3}{4}\left(B^{\prime }-4\right)\left[{\left(\frac{V_{0}}{V}\right)}^{\frac{2}{3}}-1\right]\right\},$$

with $V_{0}$ the unit-cell volume, the bulk modulus *B* and its pressure derivative $B^{\prime }$, all at zero pressure. Figure 3c displays the results and affirms the high quality of the fits. We obtained $V_{0}=\left(641.44\pm 0.3\right)\;$Å${}^{3}$ and $V_{0}=\left(638.5\pm 0.6\right)$ Å${}^{3}$ for LaPt${}_{4}$Ge${}_{12}$ and PrPt${}_{4}$Ge${}_{12}$, respectively. These values agree well with the experimental unit-cell volume at ambient pressure within the error-bars [28]. We further obtained the bulk modulus and its pressure derivative for LaPt${}_{4}$Ge${}_{12}$, $B_{0}=\left(106\pm 5\right)$ GPa and $B^{\prime }=\left(14\pm 2\right)$, and for PrPt${}_{4}$Ge${}_{12}$, $B_{0}=\left(83\pm 8\right)$ GPa and $B^{\prime }=\left(23\pm 4\right)$. We note that our experimental value for the bulk modulus of LaPt${}_{4}$Ge${}_{12}$ compares reasonably well with results from a density functional theory calculation by Tütüncü et al. [35]. There are no calculations available for PrPt${}_{4}$Ge${}_{12}$.

## 4. Discussion

Replacement of La by the smaller Pr in La${}_{1-x}$Pr${}_{x}$Pt${}_{4}$Ge${}_{12}$ results in a reduction of the lattice parameter *a* and correspondingly of the volume of the unit cell $V=a^{3}$ [3,12,25]. The unit-cell volume of PrPt${}_{4}$Ge${}_{12}$ is about 0.43% smaller than that of LaPt${}_{4}$Ge${}_{12}$ [28]. Therefore, in a simple picture, PrPt${}_{4}$Ge${}_{12}$ can be considered as a chemically pressurized analog of LaPt${}_{4}$Ge${}_{12}$, since the Pr-ion in PrPt${}_{4}$Ge${}_{12}$ is in a non-magnetic singlet crystalline electric field ground state [3,9]. In particular, no 4f magnetism competes with superconductivity [12]. Upon substituting Pr for La $T_{c}\left(x\right)$ decreases continuously up to x≈0.75, where a minimum develops before $T_{c}\left(x\right)$ increases again toward stoichiometric PrPt${}_{4}$Ge${}_{12}$ [12,25]. The nonmonotonous behavior of $T_{c}\left(x\right)$ and the appearance of time-reversal-breaking superconductivity in the substitution series La${}_{1-x}$Pr${}_{x}$Pt${}_{4}$Ge${}_{12}$ indicate that volume effects alone cannot explain the observed behavior, in particular, since substitution of La by Pr reduces the unit-cell volume linearly as function of *x* [12,25].

**Figure 4:**
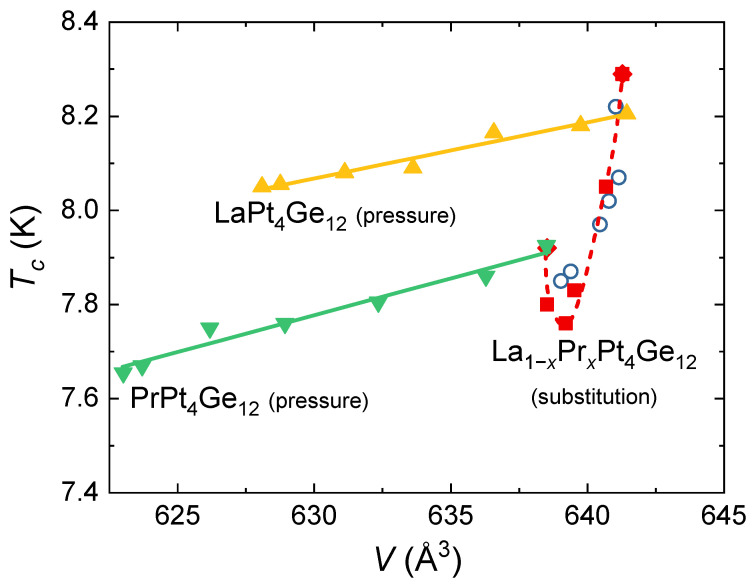
Superconducting temperature—unit-cell volume phase diagram of pressurized LaPt${}_{4}$Ge${}_{12}$ and PrPt${}_{4}$Ge${}_{12}$ combined with data of the substitution series La${}_{1-x}$Pr${}_{x}$Pt${}_{4}$Ge${}_{12}$. The open blue symbols correspond to data determined from specific heat [25], while the solid red diamonds [3] and the solid red squares [12] represent our previously published data based on magnetic susceptibility measurements taken in a magnetic field of $\mu_{0}H=2$ mT. The straight lines are fits to the data and the red dashed line is a guide to the eye.

Using the results from our XRD study, we can calculate the unit-cell volume corresponding to the applied hydrostatic pressure in our electrical-transport experiments. Figure 4 presents the resulting *T* – *V* phase diagram. It combines the results of our pressure study on LaPt${}_{4}$Ge${}_{12}$ and PrPt${}_{4}$Ge${}_{12}$ with literature data on the substitution series La${}_{1-x}$Pr${}_{x}$Pt${}_{4}$Ge${}_{12}$ [3,12,25]. We note that the structural parameters in our study as well as the ones in the studies on the substitution series were determined at room temperature. Since the thermal contraction of LaPt${}_{4}$Ge${}_{12}$ and PrPt${}_{4}$Ge${}_{12}$ is very similar this does not affect the discussion below [28]. The superconducting transition temperatures in LaPt${}_{4}$Ge${}_{12}$ and PrPt${}_{4}$Ge${}_{12}$ depend in a simple linear relation on the unit-cell volume. From fits to the data, we obtain comparable slopes of $dT_{c}\left(V\right)/dV=-11.93$ mK/Å${}^{3}$ and $dT_{c}\left(V\right)/dV=-15.65$ mK/Å${}^{3}$ for LaPt${}_{4}$Ge${}_{12}$ and PrPt${}_{4}$Ge${}_{12}$, respectively (see Figure 4). We note that due to the different bulk moduli of the two materials, the difference in $dT_{c}\left(V\right)/dV$ between LaPt${}_{4}$Ge${}_{12}$ and PrPt${}_{4}$Ge${}_{12}$ appears to be considerably smaller than the difference in the pressure dependence $dT_{c}\left(p\right)/dp$. In contrast to the simple linear and weak dependence of $T_{c}$ on the unit-cell volume in LaPt${}_{4}$Ge${}_{12}$ and PrPt${}_{4}$Ge${}_{12}$, $T_{c}\left(V\right)$ display a much stronger and non-monotonous volume dependence in the substitution series La${}_{1-x}$Pr${}_{x}$Pt${}_{4}$Ge${}_{12}$, as can be seen in Figure 4.

Fermi-surface studies on LaPt${}_{4}$Ge${}_{12}$ and PrPt${}_{4}$Ge${}_{12}$ find that their electronic band structures are nearly identical with moderately enhanced effective masses for the six bands crossing at the Fermi energy [22]. These findings are in line with phonon mediated Cooper pairing and consistent with multi-gap superconductivity in both compounds [22]. A phonon mediated superconducting coupling mechanism has been indeed suggested for LaPt${}_{4}$Ge${}_{12}$ [35]. It is therefore not surprising that $T_{c}$ exhibits the same dependence on *V* in LaPt${}_{4}$Ge${}_{12}$ and PrPt${}_{4}$Ge${}_{12}$. The similarities in the band structure are further in agreement with the continuous evolution of $T_{c}$ in the substitution series. However, $T_{c}\left(V\right)$ in the substitution series La${}_{1-x}$Pr${}_{x}$Pt${}_{4}$Ge${}_{12}$ displays a non-monotonous behavior with a pronounced minimum. Furthermore, there is evidence for time-reversal-symmetry breaking superconductivity in the substitution series, which is absent on the La-rich side [25]. We may therefore speculate that in LaPt${}_{4}$Ge${}_{12}$ and PrPt${}_{4}$Ge${}_{12}$, application of pressure in the investigated pressure range leads to a rigid shift of the band structure, similar to the findings in BaPt${}_{4-x}$Au${}_{x}$Ge${}_{12}$, where a linear change in $T_{c}\left(V\right)$ has been observed before and related to a rigid shift of the electronic band structure [36,37]. In contrast, Pr substitution in La${}_{1-x}$Pr${}_{x}$Pt${}_{4}$Ge${}_{12}$ generates distinct changes in the multi-band nature of the superconductivity, which goes beyond this simple picture and deserves further investigations.

## 5. Summary and Conclusions

We carried out electrical resistivity and X-ray diffraction experiments under hydrostatic pressures on the two skutterudite compounds, LaPt${}_{4}$Ge${}_{12}$ and PrPt${}_{4}$Ge${}_{12}$. We find a pressure-induced linear suppression of the superconduction transition temperature in both materials. Based on our XRD data, we derive a bulk modulus of 106 GPa for LaPt${}_{4}$Ge${}_{12}$ and 83 GPa for PrPt${}_{4}$Ge${}_{12}$. With the help of the bulk modulus, we established a superconducting temperature–unit-cell volume phase diagram by combining the pressure data on LaPt${}_{4}$Ge${}_{12}$ and PrPt${}_{4}$Ge${}_{12}$ with results from the substitution series La${}_{1-x}$Pr${}_{x}$Pt${}_{4}$Ge${}_{12}$. The comparison of the effect of hydrostatic pressure on LaPt${}_{4}$Ge${}_{12}$ and PrPt${}_{4}$Ge${}_{12}$ with that of chemical substitution indicates marked differences. While the weak linear dependence of $T_{c}$ on the unit-cell volume with almost the same slopes for both compounds can be explained in a simple picture consistent with phonon mediated superconductivity, the nonmonotonous dependence of $T_{c}\left(x\right)$ in La${}_{1-x}$Pr${}_{x}$Pt${}_{4}$Ge${}_{12}$ suggests more complex competing behaviors in the substitution series, which stimulate further detailed investigations.

## Data Availability

The data presented in this study are available on reasonable request from the corresponding author.
